# Evaluating the one-time chair stand test for predicting the coronavirus disease severity in patients during hospital admission: a cohort study in Japan

**DOI:** 10.1186/s12245-023-00497-x

**Published:** 2023-04-06

**Authors:** Atsushi Ishihara, Takashi Yoshizane, Teruki Mori, Yui Sasaki, Takahiro Hosokawa, Jun Suzuki, Akifumi Tsuzuku, Fumihiro Asano, Toshiyuki Noda

**Affiliations:** 1 Department of Respiratory Support Center, Gifu Prefectural General Medical Center, 500-8717 4-6-1 Noisshiki, Gifu-Shi, Gifu, 500-8717 Japan; 2 Department of Cardiology, Gifu Prefectural General Medical Center, Gifu, Japan; 3 Department of Respiratory Medicine, Gifu Prefectural General Medical Center, Gifu, Japan; 4 Department of Infectious Diseases, Gifu Prefectural General Medical Center, Gifu, Japan

**Keywords:** One-time chair stand test, COVID-19, Exercise-induced hypoxemia, Severity prediction

## Abstract

**Background:**

This study aimed to understand whether the one-time chair stand test (CS-1) is useful for predicting the severity of coronavirus disease (COVID-19) in 101 patients admitted to the hospital with acute respiratory failure.

**Methods:**

This single-centered, prospective observational cohort study enrolled 101 critically ill adult patients hospitalized with COVID-19 who underwent the CS-1 as a dynamic evaluation tool in clinical practice between late April 2020 and October 2021. Data on demographic characteristics, symptoms, laboratory values, computed tomography findings, and clinical course after admission were collected. Furthermore, the data was compared, and the association between the intubation and non-intubation groups was determined. We also calculated the cutoff point, area under the curve (AUC), and 95% confidence interval (CI) of the change in oxygen saturation (ΔSpO_2_) during the CS-1.

**Results:**

Thirty-three out of 101 patients (33%) were intubated during hospitalization. There was no significant difference in the resting SpO_2_ (93.3% versus 95.2%, *P* = 0.22), but there was a significant difference in ΔSpO_2_ during the CS-1 between the intubation and non-intubation groups (10.8% versus 5.5%, *P* < 0.01). In addition, there was a significant correlation between hospitalization and ΔSpO_2_ during the CS-1 (*ρ* = 0.60, *P* < 0.01). The generated cutoff point was calculated as 9.5% (AUC = 0.94, 95% CI = 0.88–1.00).

**Conclusion:**

For COVID-19 patients with acute respiratory failure, the CS-1 performed on admission was useful for predicting the severity of COVID-19. Furthermore, the CS-1 can be utilized as a remote and simple evaluation parameter. Thus, it could have potential clinical applications in the future.

## Background

The coronavirus disease (COVID-19) pandemic began in December 2019. Predicting decreased oxygenation in patients with COVID-19 has been difficult, and this condition indicates hospitalization for intubation and artificial respiration management [1, 2].

Studies on severity prediction have reported the usefulness of Klebs von den Lungen-6 and ferritin levels in the blood [3, 4]. However, the result from drawn blood has a time lag until the revelation. In addition, it has been reported that droplet, contact, and aerosol transmission are sources of COVID-19 [5, 6]. Therefore, a quick and efficient technique is needed to predict the severity of patients with COVID-19 requiring isolation.

In a previous report, pulmonary lesions owing to COVID-19 were reported to be similar to the condition of patients with pulmonary fibrosis [7]. Because hypoxemia occurred during exertion evaluation rather than during the resting period in patients with COVID-19 [8], we believed that the measurement of oxygen saturation (SpO_2_) at the time of the exertion evaluation was important for predicting the severity and determining the disease pathology. However, the 6-min walk test (6MWT), which is used as an exertion evaluation tool, needs a lot of space [9]. Therefore, this might not be suitable from the viewpoint of infection management. Thus, we considered the 30-s chair stand test (CS-30), which has been reported to be useful for evaluating hypoxemia, and subsequently decided to use a safer one-time chair stand test (CS-1) as an exertion evaluation tool [7, 10, 11]. This evaluation method is easy to perform; patients just have to rise up once from a chair of general height. The possibility of a remote evaluation and the requirement of less space are advantages of this examination for patients with COVID-19.

## Methods

This study aimed to understand whether CS-1 is useful for predicting the severity of COVID-19 in patients admitted to the hospital with acute respiratory failure.

### Ethics statements

The identity of patients was kept confidential, and we disclosed information about our study on our hospital’s homepage. This study received approval from the Gifu Prefectural General Medical Center Ethical Review Board (approval number: 565). All patients signed informed consent and agreed to have their anonymized clinical and investigative data used for research purposes.

### Study population

**Fig. 1:**
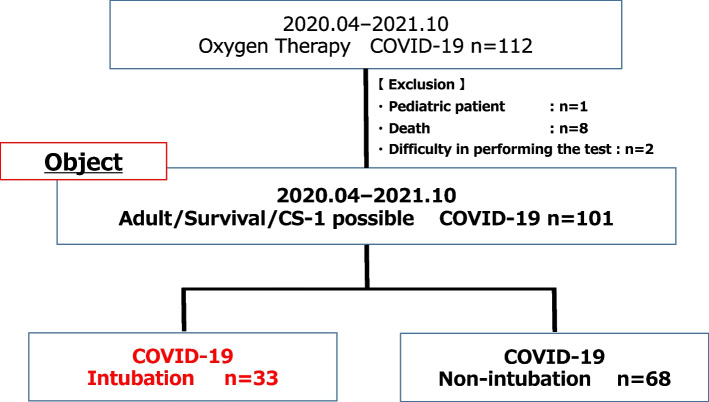
Flow diagram of patient selection. COVID-19, coronavirus disease; CS-1, one-time chair stand test

In this single-center, prospective observational cohort study, 112 patients were recruited consecutively based on COVID-19 hospital admissions with acute respiratory failure at the Gifu Prefectural General Medical Center in Japan between late April 2020 and October 2021. This study enrolled 101 critically ill adult patients with COVID-19 who underwent the CS-1 as a dynamic evaluation tool in clinical practice after excluding a pediatric patient (*n* = 1), patients who died (*n* = 8), and patients who had difficulty performing the CS-1 (*n* = 2). We assessed correct and incorrect performances of the CS-1 with a multidisciplinary team, and eight patients did not perform the CS-1 owing to death (6 patients) or because it was too difficult to perform (2 patients) (Fig. 1).

### Data collection

Patient characteristics, laboratory values, comorbidities, CS-1 results, computed tomography (CT) findings, and clinical courses after admission were collected. Additionally, CT images were evaluated by the COVID-19 team (more than two people) to determine the CT scan severity score (CTSS) [12].

**Fig. 2:**
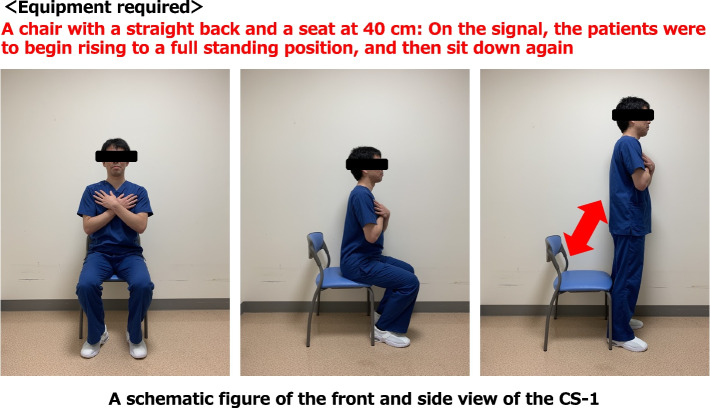
One-time chair stand test. CS-1, one-time chair stand test

The CS-1 was performed using a chair with a straight back and seat height of 40 cm; the patient was given a signal to begin rising to a full standing position and then to sit down again. A physical therapist who exclusively performed COVID-19 duties conducted this evaluation with reference to the CS-30 [13]. The patient was monitored and evaluated using a central monitor. The CS-1 results (resting SpO_2_, lowest SpO_2_, and ΔSpO_2_) were recorded during admission. In addition, SpO_2_ was measured after the CS-1, and it was continually measured until it became − 1% for resting SpO_2_. It was considered harmful if a patient took longer than 2 min to complete the CS-1. We conducted the CS-1 to quickly confirm the initial change in SpO_2_ at a safe distance from the patient for infection management purposes. The reviewers followed infection measure guidelines and wore personal protective equipment (goggle or face shield, gloves, long-sleeve gown, and hat) (Fig. 2) [5].

**Fig. 3:**
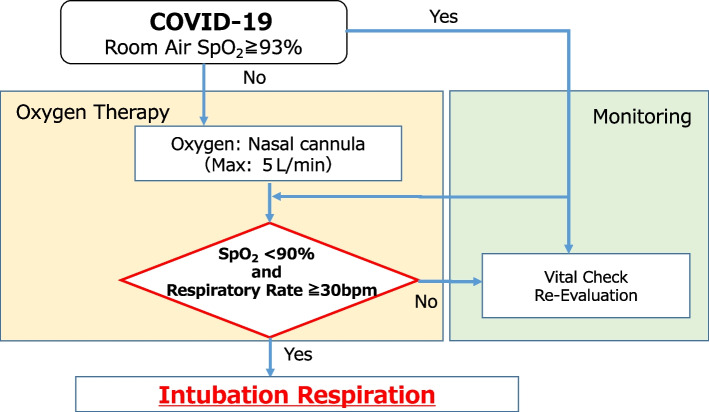
Intubation criteria for patients with COVID-19. COVID-19, coronavirus disease; SpO_2_, oxygen saturation

The correct and incorrect performances of the CS-1 were assessed, and patients who had difficulty performing it or could not perform it correctly owing to orthopedic surgery/vascular brain disease, cognitive decline, or unwillingness were excluded. The criteria for intubation on the Japanese guideline were as follows: the oxygen dose was increased from 1 to 5 L/min with a maintenance target of SpO_2_ ≥ 90% and respiratory rate being < 30 breaths/min when resting SpO_2_ was difficult to maintain at 93%, and the maintenance target could not be maintained with oxygen at 5 L/min via a cannula (Fig. 3) [14].

### Statistical analysis

Data are reported as number (%) for categorical variables and average ± standard deviation for continuous variables. First, we analyzed patient background characteristics and their association with intubation or non-intubation using the Wilcoxon rank-sum test for non-parametric continuous variables. Second, we analyzed significant correlations between hypoxemia on exertion during the CS-1 and hospitalization using the Spearman rank correlation test. Finally, we generated a receiver operating characteristic (ROC) curve of ΔSpO_2_ during the CS-1 and its association with intubation or non-intubation and calculated the cutoff point, area under the curve (AUC), and 95% confidence interval (CI) of ΔSpO_2_ during the CS-1 using the Youden Index. Two-tailed *P*-values < 0.01 were considered statistically significant. Statistical analyses were performed using the SPSS version 20 software (IBM Corp.).

## Results

A flow diagram of patient selection is shown in Fig. 1. This study prospectively investigated the relationship between ΔSpO_2_ during the CS-1 on admission and the association with intubation or non-intubation during hospitalization in 101 COVID-19 patients with acute respiratory failure. Thirty-three patients (33%) were intubated during hospitalization (Fig. 1).

Patient characteristics, laboratory values, comorbidities, CS-1 results, CT findings, and clinical courses after admission are shown on the left side in Table 1. The patients’ average age was 58.5 ± 16.7 years, 69 patients (68%) were men, and the smoking rate was 54% (54 patients). Laboratory values on admission were as follows: serum lactate dehydrogenase (LDH), 369.9 ± 166.5 IU/L; brain natriuretic hormone (BNP), 28.7 ± 38.2 pg/mL; C-reactive protein (CRP), 6.0 ± 4.9 mg/dL; and ferritin, 694.3 ± 492.7 ng/mL. CS-1 results on admission included the resting SpO_2_ at 94.6 ± 2.1%, lowest SpO_2_ at 86.5 ± 9.8%, and ΔSpO_2_ of 7.3 ± 3.4%. Furthermore, we did not observe any harm caused by the CS-1. The CTSS on admission was 6.5 ± 3.4. Clinical courses of patients after admission were hospitalization (23.8 ± 12.9 days) and other outcomes (discharge to home: 81 patients [80%]; transfer to hospital: 20 patients [20%]). Comparisons of patient background characteristics between the intubation and non-intubation groups are shown the right side in Table 1. There was no significance difference in the resting SpO_2_. The variables with a significant difference were the LDH level, lymphocyte count, ferritin level, lowest SpO_2_, ΔSpO_2_ during the CS-1, and CTSS on admission according to the Wilcoxon rank-sum test (Table 1).

**Table 1:** Comparison of background characteristics and clinical courses between the intubation and non-intubation groups

	Total (*n* = 101)	Intubation (*n* = 33)	Non-intubation (*n* = 68)	*P*-value
**Background (admission)**				
Age, years	58.5 ± 16.7	58.9 ± 12.0	58.3 ± 18.7	0.125
Sex, male	69 (68%)	24 (73%)	45 (66%)	0.513
Cigarette smoking	54 (54%)	19 (58%)	35 (52%)	0.735
BMI, kg/m^2^	25.0 ± 4.4	25.9 ± 3.9	24.5 ± 4.6	0.082
**Comorbidities**				
Hypertension	35 (35%)	15 (46%)	20 (29%)	0.513
Diabetes	24 (24%)	13 (39%)	11 (16%)	0.122
COPD	9 (9%)	4 (12%)	5 (7%)	0.782
Cardiovascular disease	9 (9%)	2 (6%)	7 (10%)	0.182
Renal failure	12 (12%)	5 (15%)	7 (10%)	0.739
Cancer	11 (11%)	3 (9%)	8 (12%)	0.157
**Laboratory (admission)**				
LDH, IU/L	369.9 ± 166.5	486.5 ± 196.5	313.3 ± 113.8	< 0.01
CRP, mg/dL	6.0 ± 4.9	8.6 ± 6.2	4.7 ± 3.6	0.130
Lymphocyte, %	15.7 ± 8.1	10.6 ± 5.7	18.2 ± 8.0	< 0.01
BNP, pg/mL	28.7 ± 38.2	34.4 ± 35.1	26.0 ± 39.6	0.797
Ferritin, ng/mL	694.3 ± 492.7	1076.0 ± 506.5	500.6 ± 355.5	< 0.01
KL-6, U/mL	356.5 ± 166.5	453.2 ± 306.8	311.2 ± 185.5	0.113
d-dimer, μg/mL	1.9 ± 2.9	3.0 ± 4.7	1.3 ± 1.0	0.052
**Image (admission)**				
CTSS	6.5 ± 3.4	9.8 ± 2.9	4.9 ± 2.4	< 0.01
**One-time chair stand test (admission)**				
Resting SpO_2_, %	94.6 ± 2.1	93.3 ± 2.6	95.2 ± 1.4	0.072
Lowest SpO_2_, %	86.5 ± 9.8	80.1 ± 14.6	89.7 ± 3.3	< 0.01
ΔSpO_2_, %	7.3 ± 3.4	10.8 ± 2.4	5.5 ± 2.3	< 0.01
**Outcome after admission**				
Hospitalization, day	23.8 ± 12.9	35.6 ± 13.7	18.1 ± 7.6	< 0.01
Return to home	81 (80%)	22 (67%)	59 (87%)	0.113

Data are presented as average ± standard deviation or number (%). Wilcoxon rank-sum test

*BMI* Body mass index, *COPD* Chronic obstructive pulmonary disease, *LDH* Serum lactate dehydrogenase, *CRP* C-reactive protein, *KL-6* Klebs von den Lungen-6, *BNP* Brain natriuretic peptide, *CTSS* Computed tomography severity score, *SpO*_*2*_ oxygen saturation

※ ΔSpO_2_ = (resting SpO_2_) − (lowest SpO_2_ on exertion)

**Fig. 4:**
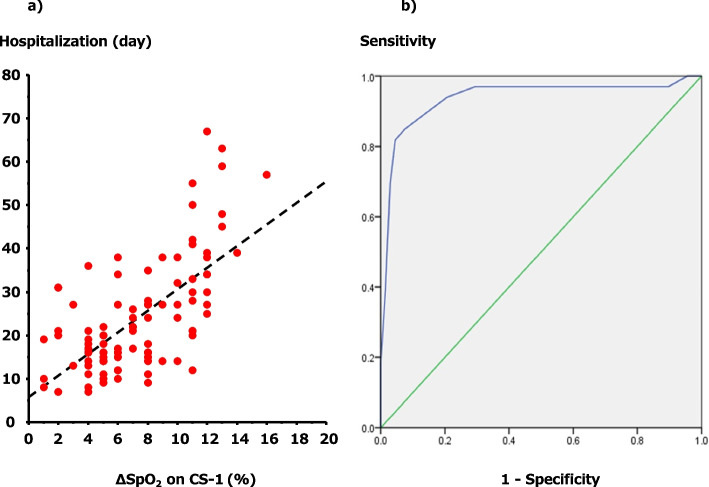
Significant correlations and cutoff points. **a** Correlation between hypoxemia on exertion during the CS-1 and hospitalization. Spearman rank correlation test, *ρ* = 0.60, *P* < 0.01. **b** Receiver operating characteristic curve for ΔSpO_2_ during the CS-1. AUC = 0.94, 95% CI = 0.88–1.00, *P* < 0.01. Cutoff point of ΔSpO_2_ during the CS-1 = 9.5%. CS-1, one-time chair stand test; SpO_2_, oxygen saturation

Significant correlations between hospitalization and ΔSpO_2_ during the CS-1, and the cutoff points of ΔSpO_2_ during the CS-1 between the intubation and non-intubation groups are shown in Fig. 4a, b. Initially, we analyzed ΔSpO_2_ during the CS-1 and its correlation with hospitalization using the Spearman rank correlation test, and we detected a meaningful equilateral correlation (*ρ* = 0.60, *P* < 0.01). Then, we generated the ROC curve for ΔSpO_2_ during the CS-1 according to intubation or non-intubation, and it showed an AUC of 0.94, 95% CI of 0.88–1.00, and cutoff point of 9.5% (Fig. 4a, b).

## Discussion

The advantages of the CS-1 used in the present study were that it required limited space, could be conducted within a short time, and made real-time dynamic evaluation possible. Based on the results of the CS-1 on admission in COVID-19 patients with acute respiratory failure, the main findings of this study are as follows. First, there was a significant difference in ΔSpO_2_ during the CS-1 on admission between the intubation and non-intubation groups. Second, there was a significant correlation between hospitalization and ΔSpO_2_ during the CS-1. Finally, the cutoff point for ΔSpO_2_ among intubated patients was 9.5%.

At first, we examined the relationship between intubation or non-intubation and SpO_2_. There was no significant difference in the resting SpO_2_. In addition to the significant difference in ΔSpO_2_ during the CS-1 between the intubation and non-intubation groups in this study, the LDH level, lymphocyte count, ferritin level, CTSS on admission, and lowest SpO_2_ during the CS-1 on admission were also significantly different between the groups. The CS-1 could be used to evaluate a patient on admission quickly and conveniently; hence, the versatility of this evaluation will make it useful in clinical practice. The CS-1 involves a compound movement and was assumed to be useful in the detection of COVID-19 severity, which is a systemic disease with various degrees of severity [15]. We believed that the CS-1 is an efficient evaluation technique compared to the CS-30 because it could be performed while maintaining social distance to ensure infection management for patients with COVID-19. In addition to ΔSpO_2_, the CS-1 will be useful in determining intubation requirement in clinical practice.

Next, we observed a significant correlation between hospitalization and ΔSpO_2_ during the CS-1. There have been some reports about exercise-induced hypoxemia. Based on them, we considered the effect of ventilation disorder caused by pulmonary alveolus diffusion disturbance and the presence of a pulmonary infiltration shadow in both lungs, which is associated with COVID-19, mentioned for the first time in this study [7]. Mason et al. suggests that there is an increase in type II alveolus epithelium cells because of a surfactant factor induced by COVID-19, causing a pulmonary diffusing capacity disorder, and this condition partially resembles pulmonary fibrosis [16]. The average CTSS on admission was 6.5 in this study, supporting the existence of pulmonary lesions and might reflect a diffusing capacity disorder. One of the disease severity indexes for pulmonary fibrosis is exercise-induced hypoxemia, where the 6MWT has been widely used as an exercise evaluation tool. Additionally, some studies showed a significant correlation between hypoxemia during the 6MWT and CS-30 in patients with interstitial lung disease [8, 11]. Because the CS-1 used in this study involved the same single rising movement as in the CS-30, we considered the detection of a similar significant decrease in SpO_2_ on exertion. Generally, necessary treatment increased along with disease severity during hospitalization, and our findings showed a significant correlation between hospitalization and ΔSpO_2_. It is interesting from the viewpoint of early treatment intervention and bed control that the CS-1 results on admission can predict hospitalization. Furthermore, the CS-1 may be used as a hospitalization criterion for patients with COVID-19 in the outpatient department, and it may be a useful remote rating system when new infectious diseases occur in the future.

Finally, we examined the cutoff point of ΔSpO_2_ among intubated patients. Attention to severity prediction and aggravation of hospitalization is needed in intubated patients with ΔSpO_2_ of 9.5% or more. This cutoff point may be useful for predicting hospitalization and disease severity in patients with COVID-19, for whom contact is restricted owing to infection management, and the CS-1 is accurate and convenient to perform during admission [5, 6]. Exploring patients’ respiratory conditions and determining the curative effect of treatment will be important when assessing ΔSpO_2_ in the future as well. This study has some limitations as follows. First, the CS-1, used as an exertion evaluation tool is a rating system devised based on the CS-30. Thus, there is no previous report to validate the usefulness of the CS-1 for COVID-19. Furthermore, we discussed the multidisciplinary use and correct and incorrect performances of the CS-1; eight patients did not perform the CS-1 in this study. The patients with COVID-19 who performed the CS-1 were not affected and could be evaluated safely, but there is no established selection protocol. Of note, two patients who completed the CS-1 died during hospitalization. The more general 6MWT has been used as a rating system, but we used the CS-1 because it provided a result after light exertion by the patient and could be performed with social distancing; this was beneficial considering infection management for COVID-19. We will examine the immediate effect of the CS-1 in future studies. In addition, because COVID-19 is like pulmonary fibrosis, the CS-1 could also be used for other diseases such as interstitial lung disease. Second, we mainly considered an alveolus diffusing capacity disorder as the condition of a patient with exercise-induced hypoxemia in this study; however, the possibility of platypnea-orthodeoxia syndrome, including right-to-left shunting affecting disease severity has also been reported [17, 18]. This is a future research topic because a plural pathologic examination was not performed in this study. Third, severe acute respiratory syndrome coronavirus 2 mutated over the 1.5 years during this study. However, the CS-1 results were relatable to the disease severity, and the CS-1 was considered adaptive to many variants of COVID-19. Therefore, we may be able to use the CS-1 for COVID-19, particularly in cases where the virus mutates, and for new infectious diseases in the future. Finally, the intubation criteria set it based on the Japanese guideline between late April 2020 and October 2021. In this study, it should be considered that the criteria for intubation are strict.

## Conclusions

CS-1 performed on COVID-19 patients with acute respiratory failure on hospital admission was useful for predicting severity. Furthermore, the CS-1 could be performed as a remote and simple evaluation technique; thus, it can be used in clinical practice. In the future, the CS-1 might become an evaluation tool that can be frequently used for patients with a new variant of COVID-19 and other emerging infectious diseases.

## Data Availability

Data sharing is not applicable to this article as no datasets were generated or analyzed during the current study.

## Abbreviations

6MWT: 6-Min walk test
AUC: Area under the curve
CI: Confidence interval
COVID-19: Coronavirus disease
CS-1: One-time chair stand test
CT: Computed tomography
CTSS: CT scan severity score
LDH: Serum lactate dehydrogenase
BNP: Brain natriuretic hormone
ROC: Receiver operating characteristic
SpO_2_: Oxygen saturation
